# Comprehensive Analysis of Risk Factors for Bronchopulmonary Dysplasia in Preterm Infants in Taiwan: A Four-Year Study

**DOI:** 10.3390/children10111822

**Published:** 2023-11-17

**Authors:** Lin-Yi Huang, Ting-I Lin, Chyi-Her Lin, San-Nan Yang, Wan-Ju Chen, Chien-Yi Wu, Hsien-Kuan Liu, Pei-Ling Wu, Jau-Ling Suen, Jung-Sheng Chen, Yung-Ning Yang

**Affiliations:** 1 Department of Pediatrics, E-DA Hospital, I-Shou University, Kaohsiung 82445, Taiwan; ed112737@edah.org.tw (L.-Y.H.); ed107994@edah.org.tw (T.-I.L.); neonate@mail.ncku.edu.tw (C.-H.L.); ed107078@edah.org.tw (S.-N.Y.); ed107686@edah.org.tw (W.-J.C.); ed101010@edah.org.tw (C.-Y.W.); ed106570@edah.org.tw (H.-K.L.); ed107560@edah.org.tw (P.-L.W.); 2 School of Medicine, I-Shou University, Kaohsiung 82445, Taiwan; 3 Department of Pediatrics, National Cheng-Kung University Hospital, Tainan 70403, Taiwan; 4 Department of Medical Research, Kaohsiung Medical University Hospital, Kaohsiung 80708, Taiwan; jlsuen@kmu.edu.tw; 5 Graduate Institute of Medicine, College of Medicine, Kaohsiung Medical University, Kaohsiung 80708, Taiwan; 6 Research Center for Precision Environmental Medicine, Kaohsiung Medical University, Kaohsiung 80756, Taiwan; 7 Department of Medical Research, E-Da Hospital, I-Shou University, Kaohsiung 824005, Taiwan; ed113187@edah.org.tw

**Keywords:** bronchopulmonary dysplasia, preterm infants, mortality

## Abstract

Bronchopulmonary dysplasia (BPD) is a major respiratory condition mainly affecting premature infants. Although its occurrence is global, risk factors may differ regionally. This study, involving 3111 infants with birth weight ≤ 1500 gm or gestational age (GA) < 30 weeks, aimed to identify risk factors for BPD and BPD/mortality in Taiwan using data from the Taiwan Neonatal Network. The BPD criteria were based on the National Institute of Child Health and Human Development standards. Average GA was 27.5 weeks, with 23.7% classified as small for GA (SGA). Multivariate analysis highlighted low GA, low birth weight, and other perinatal factors as significant risk indicators for BPD. For moderate-to-severe BPD, additional risk factors included male gender and SGA, endotracheal intubation (ETT) or cardiopulmonary cerebral resuscitation (CPCR) in initial resuscitation. In the moderate-to-severe BPD/death group, SGA and ETT or CPCR in initial resuscitation remained the only additional risk factors. The study pinpoints male gender, SGA and ETT or CPCR as key risk factors for moderate-to-severe BPD/death in low-birth-weight infants in Taiwan, offering a basis for focused interventions and further research.

## 1. Introduction

Bronchopulmonary dysplasia (BPD) is the most common pulmonary disorder among premature infants, posing significant healthcare challenges worldwide [1]. This condition, characterized by disrupted lung development, not only leads to immediate respiratory complications but also has profound effects on long-term respiratory function, neurodevelopmental progress, and overall growth [2]. Northway, Rosen, and Porter originally identified BPD in preterm infants subjected to high oxygen levels and non-positive end-expiratory pressure mechanical ventilation, noting its distinction from hyaline membrane disease [3]. The identification of BPD risk factors is essential due to the considerable emotional and economic impact on the families of affected infants. These risk factors range from prenatal to postnatal, with prematurity and low birth weight being the most frequently observed [4]. Infants weighing under 1500 g are at an increased risk, often necessitating extended use of mechanical ventilation and oxygen therapy [5]. Such infants are more vulnerable to respiratory distress syndrome than their full-term counterparts, leading to greater mechanical ventilation and oxygen requirements. Additional significant risk factors include male gender, intrauterine growth restriction, chorioamnionitis, maternal smoking, infections, and variations in parental race or ethnicity [4,6,7,8,9]. Moreover, genetic factors, especially in twins, have been implicated in the predisposition to BPD [9].

Histopathologically, the classic form of BPD (old BPD) presents with severe airway damage, prominent smooth muscle hyperplasia, and extensive alveolar septal fibrosis, predominantly resulting from the high levels of oxygen and aggressive ventilation historically used in treatment [3]. In contrast, the modern form of the disease (new BPD) is characterized by alveolar simplification, where alveoli are fewer and enlarged, reducing the surface area for gas exchange. Additionally, although less pronounced than in old BPD, new BPD can still display features such as increased airway smooth muscle hyperplasia and some degree of septal fibrosis [10]. Furthermore, impairments in surfactant production and abnormal vascular growth have been observed, exacerbating the condition [10,11].

The diagnostic criteria for BPD have evolved over the years, incorporating various factors to more accurately classify the disease [12]. Originating in 1967 as a description of progressive emphysema induced by oxygen toxicity and ventilator damage [3], the definition underwent significant changes. By 1988, it included the need for supplemental oxygen at 36 weeks of post-menstrual age (PMA) [13]. Further refinements were made in 2001, incorporating a 28 day post-birth oxygen requirement along with a new severity classification [14]. In 2018, the National Heart, Lung, and Blood Institute (NHLBI) workshop revised the definition, removing the 28 day oxygen supplement requirement [15]. Jensen et al., in 2019, revised the National Institute of Child Health and Human Development (NICHD) workshop definition to emphasize the use of positive pressure rather than oxygen supplementation for BPD classification at 36 weeks PMA [16].

To understand whether these risk factors similarly impact the Taiwanese population, we utilized data from the Taiwan Neonatal Network (TNN). TNN is a collaborative platform engaging neonatal intensive care units (NICUs) across Taiwan with the primary focus on enhancing neonatal care, particularly for very low-birthweight infants. Our study aims to examine the frequency of a combined outcome involving moderate-to-severe BPD and/or death and to elucidate the associated risk factors among low-birthweight premature infants in Taiwan. These insights could facilitate early identification and targeted intervention strategies to reduce BPD incidence and improve long-term outcomes. Moreover, given the evolving definitions of BPD, this study also explores whether different diagnostic criteria could influence the identification of risk factors.

## 2. Materials and Methods

### 2.1. Study Population

We conducted a retrospective cohort data collection from TNN. We incorporated data from neonates who had a birth weight (BW) of ≤1500 gm or a gestational age (GA) under 30 weeks. These data, collected between January 2016 and December 2019, span 26 participating hospitals, encompassing a majority of the secondary and tertiary NICUs in Taiwan. Newborns with major congenital anomalies were excluded. Data were recorded using a standardized electronic case report form. All methods were performed in accordance with the relevant guidelines and regulations.

### 2.2. Outcome Measures and Variables

The primary outcome measure was the diagnosis of BPD, defined as a requirement for at least 28 days of >21% oxygen. Patients were categorized into mild BPD (no need for supplemental oxygen at 36 weeks PMA) and moderate-to-severe BPD (requirement for >21% oxygen at 36 weeks PMA). Variables considered for risk factor analysis included:

Maternal factors: nationality, mode of delivery, multiple births, antenatal steroid use, magnesium sulfate use during any trimester, chorioamnionitis and pregnancy-induced hypertension (PIH).

Neonatal factors: birth weight, small/average/large for gestational age (SGA/AGA/LGA), gender, 5 minute Apgar scores, type of delivery room resuscitation, sepsis/meningitis, intraventricular hemorrhage (IVH), respiratory distress syndrome (RDS), assisted ventilator support, surfactant therapy, inhaled nitric oxide (NO) therapy, pneumothorax, patent ductus arteriosus (PDA) and necrotizing enterocolitis (NEC).

Definitions for all diagnostic criteria and support interventions are as follows: infants with a birth weight below the 10th percentile for their GA were classified as SGA, those above the 10th percentile were considered LGA, and the remainder were categorized as AGA. Assisted ventilator support included face mask ventilation, mechanical ventilation (MV), high-frequency oscillatory ventilation (HFO), high-flow nasal canula, nasal continuous positive airway pressure (nCPAP), nasal intermittent mandatory ventilation (nIMV), or nasal synchronized intermittent mandatory ventilation (nSIMV). The definitions of sepsis and meningitis were given by a proven pathogenic bacteria growth from blood culture and cerebrospinal fluid culture; the definition of IVH was grade 1–4 IVH based on cranial imaging; while the definition of RDS was to fulfil both of the below criteria within 24 h of birth: typical RDS pattern on chest X ray, and any one of the following: arterial oxygen pressure (PaO2) < 50 mmHg under room air, central cyanosis under room air, the need for assisted ventilator support to maintain PaO2 > 50 mmHg, or requiring assisted ventilator support to maintain oxygen saturation (SpO2) > 85%. The definition of PDA employed in our study required the fulfillment of two criteria: first, the detection of PDA on echocardiography, which is evidenced by a left to right shunt or a bidirectional shunt; and second, clinical signs consistent with a significant PDA, such as a hyperdynamic precordium, bounding pulses, wide pulse pressure, evidence of pulmonary vascular congestion, or cardiomegaly. The definition of NEC was to fulfil at least one clinical symptom and one image finding. Clinical symptoms included bilious vomiting, abdominal distension and bloody stool. Image findings included pneumatosis intestinalis, hepato-biliary gas and pneumoperitoneum.

### 2.3. Statistics

SPSS version 17.0 (SPSS Inc., Chicago, IL, USA) was used for all statistical analyses. The *χ*^2^ test and Fisher’s exact test were used to determine significant differences between groups with categorical variables. Univariate analyses were used to determine the association between potential risk factors of BPD. Multivariate logistic regression analysis was performed to identify independent significant risk factors for BPD. Statistical significance was set at *p* < 0.05.

## 3. Results

**Figure 1:**
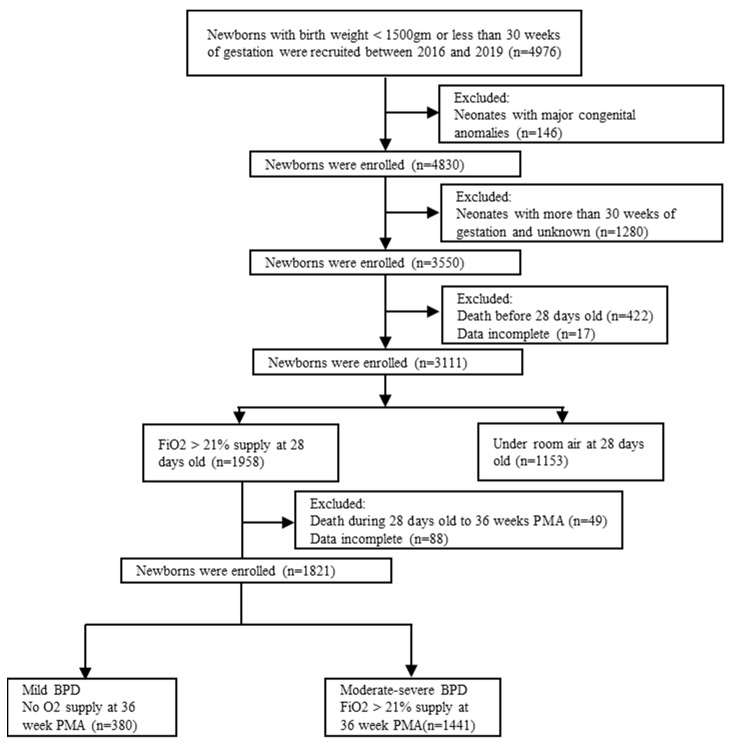
Flow chart of case enrollment. BPD, bronchopulmonary dysplasia; PMA, postmenstrual age.

A total of 3550 newborns with a BW of ≤1500 gm or a GA of less than 30 weeks were enrolled between 2016 and 2019 (Figure 1). After excluding neonates who died before 28 days of age and those with missing data, 3111 newborns were included in the study analysis. Among these, 1153 (37%) did not have BPD, 380 (12%) had mild BPD, and 1441 (46%) had moderate-to-severe BPD.

### 3.1. Demographics and Clinical Characteristics

The average GA was 27.5 weeks, with 1405 cases (45.2%) having a GA of less than 28 weeks, with the majority (1679 cases, 54%) being male. The distribution by size for GA was as follows: 738 cases (23.7%) were SGA, 2342 cases (75.3%) were AGA, and 31 cases (1.0%) were LGA. Cesarean section was the mode of delivery for 2093 cases (67.3%), and 878 cases (28.2%) were part of multiple births (Table 1).

In our investigation of BPD risk factors, we performed a comprehensive statistical analysis presented in Table 2. In univariate analyses, variables significantly correlated with BPD encompassed a range of clinical and perinatal factors: low GA, birth by normal spontaneous delivery (NSD), maternal chorioamnionitis, absence of maternal PIH, BBW ≤ 1000 g, and a suboptimal 5 minute Apgar score (≤7). Other risk-enhancing variables included the necessity for oxygenation during initial resuscitation, requirement for endotracheal intubation (ETT) or cardiopulmonary cerebral resuscitation (CPCR) in the resuscitative phase, the absence of nCPAP application initially, and RDS. Prolonged oxygen use during hospitalization, different ventilatory modalities like ETT with MV or HFO, nasal high-flow cannula or nIMV or nSIMV, the absence of nCPAP during hospitalization, surfactant administration, inhaled NO therapy, and various neonatal complications such as pneumothorax, neonatal sepsis/meningitis, IVH, PDA and NEC were also found to be significant factors. Death was an additional significant variable.

Subsequent multivariate logistic regression analysis, elaborated in Table 3, identified a subset of independent risk factors with a significance level of *p* < 0.05. These included low GA, maternal chorioamnionitis, BBW ≤ 1000 g, a 5 minute Apgar score less than 7, RDS, and requirements for various forms of assisted ventilator support during hospitalization, including oxygenation, MV, or HFO, nasal high-flow cannula, or nIMV or nSIMV. The administration of inhaled NO therapy, neonatal sepsis/meningitis and PDA were also found to be independently significant.

### 3.2. Risk Factors of Moderate-to-Severe BPD/Death

In our study, Table 4 delineates the independent risk factors for moderate-to-severe BPD compared with the non-BPD group. Our findings indicate that several variables, including low GA, maternal chorioamnionitis, BBW ≤ 1000 g, RDS, male gender, SGA and interventions such as ETT or CPCR during initial resuscitation, significantly contributed to the risk. Additionally, types of ventilatory support employed during hospitalization—oxygen therapy, MV, HFO as well as the use of high-flow nasal cannula or nIMV or nSIMV—were also identified as independent risk factors. Other clinical variables such as inhaled NO therapy, neonatal sepsis/meningitis and PDA were also found to be significant.

We further analyzed these independent risk factors within two subgroups: those with moderate-to-severe BPD/death before reaching 28 days of age, and those with moderate-to-severe BPD/death before attaining 36 weeks PMA compared with the non-BPD group. The risk profile for these subgroups mirrored that for moderate-to-severe BPD, with one notable exception. Male gender emerged as a significant risk factor exclusively in the moderate-to-severe BPD cohort, but did not retain its significance in the other two subgroups.

## 4. Discussion

This study, based on four years of data from the TNN, is among the most comprehensive in recent years for identifying risk factors associated with BPD in preterm infants in Taiwan. Utilizing multivariate logistic regression analyses, we identified a myriad of risk factors for BPD, including low GA, low birth weight, chorioamnionitis, male gender, and various clinical interventions such as intubation and oxygen therapies. Additionally, we examined factors contributing to unfavorable outcomes, such as moderate-to-severe BPD, neonatal mortality within 28 days, and mortality within 36 weeks PMA. Notably, male gender, SGA, and ETT or CPCR in initial resuscitation were additional risk factors for these severe outcomes.

The evolution of the definition of BPD has been intricately tied with the aim of more accurately predicting disease severity and associated complications. One pivotal definition came from a 2001 workshop organized by the NICHD [17] where, according to these guidelines, for infants born at less than 32 weeks of GA, BPD was defined as requiring more than 21% oxygen for at least 28 days followed by an evaluation at 36 weeks PMA or upon hospital discharge. For those born at a GA greater than 32 weeks, the criteria stated a need for less than 21% supplemental oxygen for at least 28 days, followed by an evaluation at 56 days postnatally or upon discharge [17]. This definition further categorizes BPD severity into mild, moderate, and severe categories, based on specific oxygen and respiratory support requirements at 36 weeks PMA or discharge [14].

A 2016 NICHD workshop led to subsequent updates, with results published in 2018 [15]. This revised framework included Grade I to Grade III(A) classifications, each corresponding to varying levels of oxygen and ventilatory support needs. Other additional classification criteria have come from sources such as the Canadian Neonatal Network (CNN) in 2017 and Jensen in 2019, which both incorporate various respiratory ventilation parameters [18]. Given the current lack of a consensus definition for BPD, our study opted to utilize the NICHD 2001 guidelines. Specifically, we defined BPD as requiring more than 28 days of oxygen therapy. For severity stratification, we adhered to the NICHD 2001 criteria: mild BPD was defined as no supplemental oxygen requirement at 36 weeks PMA, while severe BPD required an FiO2 greater than 21% [17]. The primary objective of our study was to identify risk factors that significantly influenced the prognosis of BPD within the Taiwanese neonatal population [19].

In the realm of extremely low GA prematurity, mortality and BPD are often considered competing outcomes. Hence, many studies define composite outcomes encompassing either BPD or death, with some even categorizing death as an indicator of severe BPD in order to probe the associated risk factors [20,21,22]. Our investigation chose to include death in the composite outcomes for moderate-to-severe BPD, specifically for cases occurring within 28 days and 36 weeks PMA.

In our study, multiple logistic regression analysis identified a spectrum of risk factors for BPD that included GA, chorioamnionitis, PIH, low birth bodyweight, low APGAR score at birth, gender, oxygen usage during hospitalization, and various modes of ventilation, sepsis and PDA. Our findings are in line with earlier studies emphasizing the significant roles of low GA and reduced birthweight in elevating the risk for BPD [22,23]. Notably, our data reveal statistically significant associations in each of the examined groups (Table 4). Other factors are similar to those in other reports that take even death in 28 days or 36 weeks into the calculation.

However, a point of divergence arises concerning the gender variable. In our study, the male gender was identified as a significant risk factor specifically for moderate-to-severe BPD/death. Interestingly, no significant gender differentiation was evident in cases where BPD was associated with death within 36 weeks (*p* = 0.062). Previous literature on the role of male gender as a risk factor for BPD has been inconsistent; some studies have identified it as a risk factor [24], whereas others have found no consistent relationship between male gender and adverse BPD outcomes [22,25,26].

These disparities necessitate further research in order to unearth the underlying mechanisms potentially accounting for the observed gender-based risk differences for BPD. Future investigations may delve into possible biological, hormonal, or even socio-environmental factors that could mediate these gender-specific risks. Furthermore, another additional risk factor for moderate-to-severe BPD/death in this study was SGA. SGA was reported as being associated with decreased chance of survival [27]. Although a previous study revealed SGA below 32 weeks gestation was associated with neonatal mortality and also neonatal pulmonary morbidity [28], our data show it was only a risk factor in the moderate-to-severe BPD/death group.

One surprising finding was the statistical significance of a low APGAR score (<7) at 5 min in the BPD group but not in moderate-to-severe BPD/death groups. The impact of a low Apgar score on the development of BPD has yielded inconsistent results across studies [29,30], with the role of a low Apgar score as a risk factor for moderate-to-severe BPD/death remaining inconclusive. However, ETT or CPCR in initial resuscitation was statistical significant in moderate-to-severe BPD/death groups but not in the BPD group. In preterm infants, the use of ETT or CPCR in initial resuscitation may be more indicative of their birth condition and the subsequent severity of BPD than the low APGAR score. This area may warrant further investigation in order to elucidate the underlying reasons for these discrepancies.

This study’s strength is augmented by its comprehensive dataset, encapsulating nearly all instances of preterm births across Taiwan. This makes it one of the most geographically and demographically representative studies on the subject within the region. Nonetheless, the retrospective nature of the study, coupled with the absence of cross-hospital data verification, introduces limitations that warrant careful interpretation of our findings.

## 5. Conclusions

In summary, low GA, low birth bodyweight, infection, ventilator use, and nitric oxide inhalation were all important risk factors of BPD. Additionally, the indicators of SGA, male gender, and ETT or CPCR in initial resuscitation are more inclined to be associated with the development of moderate-to-severe BPD. According to this study, future prospective research could be conducted to regulate the aforementioned risk factors and elucidate more effective methods to reduce BPD. In recent years, the TNN has amassed a substantial amount of data concerning BPD in preterm infants. It is anticipated that this will pave the way for more detailed and comprehensive studies in the future.

## Figures and Tables

**Table 1:** Clinical characteristic of enrolled newborn case.

Total (N = 3111)	N (%)
**Gestational age, Mean ± SD**	27.5 ± 2.0
<28 week	1405 (45.2)
≥28 week	1706 (54.8)
Sex	
Male	1679 (54.0)
Female	1432 (46.0)
**SGA/AGA/LGA**	
Small for gestational age	738 (23.7)
Appropriate for gestational age	2342 (75.3)
Large for gestational age	31 (1.0)
**Birth weight (g)**	
<501	51 (1.6)
501–600	122 (3.9)
601–700	281 (9.0)
701–800	321 (10.3)
801–900	364 (11.7)
901–1000	360 (11.6)
1001–1100	355 (11.4)
1101–1200	340 (10.9)
1201–1300	293 (9.4)
1301–1400	314 (10.1)
1401–1500	249 (8.0)
>1500 g	61 (2.0)
**Delivery**	
Vaginal delivery	999 (32.1)
Cesarean section	2093 (67.3)
**Multiple births**	
No	2233 (71.8)
Yes	878 (28.2)

**Table 2:** Risk factor analysis between BPD and non-BPD group.

N = 3111	Non-BPD (n = 1153)	BPD (n = 1958)	*p* Value
GA, mean ± SD	28.70 ± 1.235	26.76 ± 1.974	<0.001 ***
Taiwan nationality, n (%)	1123 (37.2)	1898 (62.8)	0.265
Multiple births, n (%)	336 (38.3)	542 (61.7)	0.202
NSD, n (%)	330 (33.0)	669 (67.0)	0.001 **
Maternal ANS, n (%)	997 (37.4)	1672 (62.6)	0.152
Maternal MgSO4 use, n (%)	670 (37.6)	1112 (62.4)	0.242
Chorioamnionitis, n (%)	135 (23.7)	435 (76.3)	<0.001 ***
Non-PIH, n (%)	854 (35.3)	1563 (64.7)	<0.001 ***
Low BBW (≤1000 g), n (%)	274 (18.3)	1225 (81.7)	<0.001 ***
Male gender, n (%)	603 (35.9)	1076 (64.1)	0.081
BBW for GA, n (%)			0.318
SGA	290 (39.3)	448 (60.7)	
AGA	853 (36.4)	1489 (63.6)	
LGA	10 (32.3)	21 (67.7)	
5 minute Apgar score, n (%)			<0.001 ***
0–6	114 (20.8)	435 (79.2)	
7–10	1035 (40.6)	1517 (59.4)	
Oxygen or face mask use in initial resuscitation, n (%)	1069 (36.0)	1901 (64.0)	<0.001 ***
ETT or CPCR in initial resuscitation, n (%)	177 (16.1)	925 (83.9)	<0.001 ***
Non-nCPAP in initial resuscitation, n (%)	738 (34.0)	1432 (66.0)	<0.001 ***
Bacteremia before 3 days old, n (%)	31 (31.6)	67 (68.4)	0.153
RDS, n (%)	864 (34.4)	1647 (65.6)	<0.001 ***
Oxygen use during hospitalization	972 (33.3)	1951 (66.7)	<0.001 ***
ETT+MV or ETT+HFO during hospitalization, n (%)	353 (19.1)	1499 (80.9)	<0.001 ***
Nasal high-flow canula or nIMV or nSIMV during hospitalization, n (%)	741 (32.1)	1566 (67.9)	<0.001 ***
Non-nCPAP use during hospitalization	26 (20.8)	99 (79.2)	<0.001 ***
Non-nCPAP use before ETT, n (%)	139 (16.3)	714 (83.7)	<0.001 ***
Surfactant therapy in initial resuscitation, n (%)	1 (16.7)	5 (83.3)	0.281
Surfactant therapy during hospitalization, n (%)	293 (21.5)	1071 (78.5)	<0.001 ***
Inhaled NO therapy, n (%)	10 (3.6)	269 (96.4)	<0.001 ***
Pneumothorax	28 (18.7)	122 (81.3)	<0.001 ***
Sepsis or meningitis, n (%)	46 (13.3)	301 (86.7)	<0.001 ***
IVH, n (%)			<0.001 ***
0–2	1117 (39.0)	1744 (61.0)	
3–4	35 (14.3)	210 (85.7)	
PDA, n (%)	435 (25.9)	1246 (74.1)	<0.001 ***
NEC, n (%)	45 (23.8)	144 (76.2)	<0.001 ***
Death, n (%)	3 (3.1)	94 (96.9)	<0.001 ***

BPD, bronchopulmonary dysplasia; GA, gestational age; SD, standard deviation; NSD, natural spontaneous delivery; ANS, antenatal steroid; PIH, pregnancy-induced hypertension; BBW, birth body weight; SGA, small for gestational age; AGA, appropriate for gestational age; LGA, large for gestational age; ETT, endotracheal intubation; CPCR, cardiopulmonary cerebral resuscitation; RDS, respiratory distress syndrome; MV, mechanical ventilation; HFO, high-frequency oscillatory ventilation; nIMV, nasal intermittent mandatory ventilation; nSIMV, nasal synchronized intermittent mandatory ventilation; nCPAP, nasal continuous positive airway pressure; NO, nitric oxide; IVH, intraventricular hemorrhage; PDA, patent ductus arteriosus; NEC, necrotizing enterocolitis (** *p* < 0.01, *** *p* < 0.001).

**Table 3:** Multivariate logistic regression analysis of risk factor for BPD.

N = 3111	OR (95% CI)	*p* Value
Low GA	0.661 (0.604~0.723)	<0.001 ***
Multiple births	1.152 (0.921~1.440)	0.216
Cesarean section	0.813 (0.642~1.029)	0.085
Maternal ANS	0.984 (0.721~1.342)	0.919
Maternal MgSO4 use	0.839 (0.677~1.040)	0.110
Chorioamnionitis	1.781 (1.347~2.356)	<0.001 ***
Maternal PIH	0.969 (0.747~1.258)	0.814
Low BBW (≦1000 g)	1.675 (1.245~2.252)	0.001 **
Male gender	1.143 (0.941~1.387)	0.178
BBW for GA		0.681
SGA	–	
AGA	0.893 (0.666~1.197)	
LGA	0.719 (0.270~1.920)	
Five minute Apgar score		0.042 *
0–6	–	
7–10	1.398 (1.012~1.932)	
Oxygen or face mask use in initial resuscitation	1.255 (0.735~2.144)	0.405
ETT or CPCR in initial resuscitation	1.305 (0.965~1.765)	0.084
nCPAP in initial resuscitation	0.925 (0.740~1.156)	0.491
Bacteremia before 3 days old	0.651 (0.353~1.199)	0.169
RDS	0.687 (0.528~0.894)	0.005 **
Oxygen use during hospitalization	18.334 (7.951~42.275)	<0.001 ***
ETT + MV or ETT + HFO during hospitalization	2.315 (1.785~3.003)	<0.001 ***
Nasal high-flow canula or nIMV or nSIMV during hospitalization	1.762 (1.393~2.229)	<0.001 ***
nCPAP during hospitalization	0.870 (0.446~1.695)	0.682
Surfactant therapy in initial resuscitation	1.646 (0.140~19.363)	0.692
Surfactant therapy during hospitalization	1.173 (0.913~1.508)	0.212
Inhaled NO therapy	5.461 (2.531~11.783)	<0.001 ***
Pneumothorax	0.945 (0.547~1.633)	0.840
Sepsis or meningitis	2.314 (1.578~3.392)	<0.001 ***
IVH		0.480
0–2	–	
3–4	1.179 (0.746~1.865)	
PDA	1.295 (1.051~1.596)	0.015 *
NEC	0.937 (0.592~1.485)	0.783

BPD, bronchopulmonary dysplasia; GA, gestational age; ANS, antenatal steroid; PIH, pregnancy-induced hypertension; BBW, birth body weight; SGA, small for gestational age; AGA, appropriate for gestational age; LGA, large for gestational age; ETT, endotracheal intubation; CPCR, cardiopulmonary cerebral resuscitation; RDS, respiratory distress syndrome; MV, mechanical ventilation; HFO, high-frequency oscillatory ventilation; nIMV, nasal intermittent mandatory ventilation; nSIMV, nasal synchronized intermittent mandatory ventilation; nCPAP, nasal continuous positive airway pressure; NO, nitric oxide; IVH, intraventricular hemorrhage; PDA, patent ductus arteriosus; NEC, necrotizing enterocolitis. (* *p* < 0.05, ** *p* < 0.01, *** *p* < 0.001).

**Table 4:** Multivariate logistic regression analysis of risk factor for moderate-to-severe BPD; subgroup analysis of moderate-to-severe BPD/death before 28 days old and moderate-to-severe BPD/death before 36 weeks PMA.

	Moderate-to-Severe BPD n = 2594		Moderate-to-Severe BPD/Death before 28 Days Old n = 3016		Moderate-to-Severe BPD/Death before 36 Weeks PMA n = 3067	
	OR (95% CI)	*p* Value	OR (95% CI)	*p* Value	OR (95% CI)	*p* Value
Low GA	0.612 (0.551~0.679)	<0.001 ***	0.609 (0.549~0.675)	<0.001 ***	0.612 (0.552~0.678)	<0.001 ***
Multiple births	1.245 (0.956~1.620)	0.103	1.225 (0.944~1.588)	0.127	1.221 (0.942~1.583)	0.132
Cesarean section	0.801 (0.604~1.062)	0.123	0.806 (0.610~1.065)	0.129	0.794 (0.602~1.049)	0.105
Maternal ANS	1.256 (0.867~1.820)	0.228	1.189 (0.826~1.713)	0.352	1.213 (0.843~1.747)	0.298
Maternal MgSO4 use	0.812 (0.630~1.045)	0.106	0.841 (0.655~1.078)	0.172	0.839 (0.655~1.076)	0.167
Chorioamnionitis	1.686 (1.216~2.338)	0.002 **	1.642 (1.188~2.268)	0.003 **	1.613 (1.168~2.226)	0.004 **
Maternal PIH	0.950 (0.699~1.291)	0.744	0.932 (0.689~1.260)	0.646	0.931 (0.689~1.259)	0.643
Low BBW (≦1000 g)	2.049 (1.473~2.857)	<0.001 ***	1.992 (1.433~2.762)	<0.001 ***	1.992 (1.435~2.762)	<0.001 ***
Male gender	1.259 (1.002~1.582)	0.048 *	1.245 (0.994~1.560)	0.057	1.224 (0.978~1.531)	0.079
BBW for GA		0.023 *		0.016 *		0.018 **
SGA	–		–		–	
AGA	0.620 (0.441~0.871)		0.611 (0.437~0.856)		0.616 (0.440~0.861)	
LGA	0.578 (0.190~1.757)		0.545 (0.180~1.648)		0.545 (0.180~1.647)	
Five minute Apgar score		0.056		0.093		0.099
0–6	–		–		–	
7–10	1.412 (0.990~2.013)		1.349 (0.951~1.912)		1.341 (0.946~1.900)	
Oxygen or face mask use in initial resuscitation	1.343 (0.698~2.584)	0.377	1.372 (0.721~2.610)	0.336	1.377 (0.723~2.621)	0.331
ETT or CPCR in initial resuscitation	1.499 (1.074~2.091)	0.017 *	1.449 (1.042~2.014)	0.028 *	1.412 (1.016~1.963)	0.040 *
nCPAP in initial resuscitation	1.055 (0.809~1.375)	0.695	1.052 (0.809~1.370)	0.704	1.047 (0.804~1.361)	0.735
Bacteremia before 3 days old	0.526 (0.259~1.069)	0.076	0.541 (0.272~1.077)	0.808	0.605 (0.305~1.197)	0.149
RDS	0.788 (0.567~1.095)	0.156	0.777 (0.562~1.075)	0.127	0.769 (0.557~1.062)	0.111
Oxygen use during hospitalization	17.999 (5.660~57.236)	<0.001 ***	10.593 (4.508~24.895)	<0.001 ***	10.882 (4.618~25.643)	<0.001 ***
ETT + MV or ETT + HFO during hospitalization	3.097 (2.308~4.155)	<0.001 ***	3.112 (2.325~4.165)	<0.001 ***	3.219 (2.407~4.305)	<0.001 ***
Nasal high-flow canula or nIMV or nSIMV during hospitalization	2.046 (1.536~2.724)	<0.001 ***	1.866 (1.408~2.474)	<0.001 ***	1.804 (1.363~2.388)	<0.001 ***
nCPAP during hospitalization	1.437 (0.641~3.221)	0.379	0.528 (0.265~1.051)	0.069	0.506 (0.256~1.000)	0.050
Surfactant therapy in initial resuscitation	3.040 (0.237~38.932)	0.393	2.613 (0.179~38.082)	0.482	2.663 (0.184~38.517)	0.472
Surfactant therapy during hospitalization	1.156 (0.870~1.536)	0.318	1.164 (0.880~1.540)	0.287	1.143 (0.864~1.511)	0.349
Inhaled NO therapy	6.505 (2.945~14.370)	<0.001 ***	7.197 (3.306~15.669)	<0.001 ***	7.201 (3.317~15.633)	<0.001 ***
Pneumothorax	0.977 (0.540~1.771)	0.940	1.147 (0.638~2.065)	0.646	1.128 (0.628~2.025)	0.687
Sepsis/meningitis	2.651 (1.726~4.071)	<0.001 ***	2.887 (1.887~4.418)	<0.001 ***	2.855 (1.868~4.363)	<0.001 ***
IVH		0.781		0.269		0.155
0–2	–		–		–	
3–4	1.073 (0.653~1.765)		1.318 (0.808~2.152)		1.424 (0.875~2.318)	
PDA	1.589 (1.249~2.022)	<0.001 ***	1.541 (1.216~1.954)	<0.001 ***	1.556 (1.228~1.972)	<0.001 ***
NEC	0.921 (0.547~1.551)	0.758	1.064 (0.636~1.779)	0.814	1.083 (0.650~1.805)	0.759

BPD, bronchopulmonary dysplasia; GA, gestational age; ANS, antenatal steroid; PIH, pregnancy-induced hypertension; BBW, birth body weight; SGA, small for gestational age; AGA, appropriate for gestational age; LGA, large for gestational age; ETT, endotracheal intubation; CPCR, cardiopulmonary cerebral resuscitation; RDS, respiratory distress syndrome; MV, mechanical ventilation; HFO, high-frequency oscillatory ventilation; nIMV, nasal intermittent mandatory ventilation; nSIMV, nasal synchronized intermittent mandatory ventilation; nCPAP, nasal continuous positive airway pressure; NO, nitric oxide; IVH, intraventricular hemorrhage; PDA, patent ductus arteriosus; NEC, necrotizing enterocolitis. (* *p* < 0.05, ** *p* < 0.01, *** *p* < 0.001).

## Data Availability

Per the policy on database availability and application established by the Taiwan Neonatal Network (TNN), all data, despite being anonymized and stripped of identifying details, remain confidential. Access to TNN data is restricted to those individuals who are authorized for research purposes. Data related to this study can be obtained from the corresponding author upon a reasonable request.

## Abbreviations

BPD	bronchopulmonary dysplasia
PMA	post-menstrual age
TNN	Taiwan Neonatal Network
NICU	neonatal intensive care unit
BW	birth weight
GA	gestational age
SGA	small for gestational age
AGA	average for gestational age
LGA	large for gestational age
IVH	intraventricular hemorrhage
RDS	respiratory distress syndrome
NO	inhaled nitric oxide
PDA	patent ductus arteriosus
NEC	necrotizing enterocolitis
HFO	high-frequency oscillatory ventilation
nCPAP	nasal continuous positive airway pressure
nIMV	nasal intermittent mandatory ventilation
nSIMV	nasal synchronized intermittent mandatory ventilation
SpO2	oxygen saturation
NSD	normal spontaneous delivery
ETT	endotracheal intubation
CPCR	cardiopulmonary cerebral resuscitation
